# Global research trends of artificial intelligence in echocardiography (1984–2025): a bibliometric analysis

**DOI:** 10.1186/s44348-026-00106-4

**Published:** 2026-11-10

**Authors:** Lamia Al Saikhan

**Affiliations:** Department of Cardiac Technology, College of Applied Medical Sciences, Imam Abdulrahman Bin Faisal University, Dammam, Saudi Arabia

**Keywords:** Artificial intelligence, Machine learning, Bibliometrics, Echocardiography

## Abstract

**Background:**

Echocardiography has evolved substantially since its introduction in 1954. Artificial intelligence (AI) offers transformative potential for image acquisition, quantification, interpretation, and reporting. Despite growing research interest, a comprehensive evaluation of the historical trends and evolution of AI in echocardiography is lacking. The study aimed to conduct a comprehensive bibliometric analysis of the global research landscape in this field.

**Methods:**

A systematic literature search was conducted using the Scopus and Web of Science Core Collection databases for publications on AI in echocardiography from inception through November 10, 2025; no language filter was applied. Data analysis was performed using Microsoft Excel, Microsoft Access, and IBM SPSS Statistics. In addition, bibliometric mapping and data visualization were conducted.

**Results:**

Overall, 2682 publications received 35,083 citations (mean, 13.08 citations per publication), spanning 1984–2025. Original articles, conference papers, and review articles accounted for 87.4% and 98.5% of total publications and citations, respectively. Publication output remained limited until approximately 2015, rose gradually thereafter, and accelerated markedly after 2018, with similar output in 2024 and 2025. The United States led in both publication volume and total citations, followed by China and the United Kingdom. The United States also had the highest collaboration link strength, and seven of the top 10 institutions were in North America (six in the United States and one in Canada). Mayo Clinic ranked first, with 142 publications and 3604 citations, and had the earliest first indexed publication among the top institutions (1992). The journal *JACC: Cardiovascular Imaging* ranked first in both total citations (1,911) and citations per publication (36.75). “Echocardiography,” “deep learning,” “artificial intelligence,” and “machine learning” were the most frequently used terms. The thematic evolution showed continuity of algorithmic-centric themes (1984–2019) with emerging clinically oriented themes (2020–2025).

**Conclusions:**

This comprehensive bibliometric analysis highlights the rapid expansion of AI research in echocardiography, characterized by an accelerating publication output and emerging clinically oriented research topics. These findings reflect growing research interest in integrating AI into echocardiographic practice and highlight the need for future research prioritizing multicenter validation, clinical translation, and equitable real-world implementation.

**Supplementary Information:**

The online version contains supplementary material available at https://doi.org/10.1186/s44348-026-00106-4.

## Background

Echocardiography has evolved extensively since Edler and Hertz first applied ultrasound for cardiac assessment in 1954 [1]. Over time, the field has been continuously shaped by technological advances that have been gradually incorporated into routine clinical practice [2], making cardiac ultrasound studies more comprehensive and closely integrated with other diagnostic tools [3]. Consequently, echocardiography has become the most widely used modality for cardiac imaging [3].

As well as technological advances, artificial intelligence (AI) has been one of the most revolutionary and transformative developments in the modern history of echocardiography, despite origins dating back to the 1950s [4]. Increasing image complexity, expanding workloads, and growing demands for precision medicine have created new challenges that cannot be addressed by traditional methods alone, paving the way for AI-based solutions in the field of echocardiography [4–8]. AI has vast potential clinical applications, ranging from image acquisition and automated quantification of cardiac structure and function to image interpretation, automated reporting, and cardiovascular disease identification [4].

Given this extensive history of technological advancement, a systematic bibliometric evaluation of the emergence and evolution of AI in echocardiography literature is timely and imperative. Two recent bibliometric studies have examined this field using single-database Web of Science Core Collection (WoSCC) searches limited to English-language articles and reviews published up to 2024 [9, 10]. These studies provided valuable snapshots of recent research activity but did not provide a merged Scopus-WoSCC map across the full historical period of indexed AI-echocardiography literature. Therefore, this study aimed to conduct a comprehensive bibliometric analysis of AI applications in echocardiography across multiple databases and over an extended timeframe, providing a broader perspective on historical trajectories, contributors, thematic evolution, citation-associated characteristics, and emerging future research directions in this field.

The following research questions guided this bibliometric analysis: (1) how has global AI-echocardiography research evolved historically from the earliest indexed literature to 2025?; (2) which countries, institutions, journals, and collaboration networks have shaped the field?; (3) how have research themes evolved over time, and to what extent have clinically oriented topics emerged?; (4) which publication characteristics are associated with citation visibility?; and (5) what knowledge gaps and future directions are suggested by the current bibliometric landscape?

## Methods

### Data collection and search strategy

A comprehensive literature search in the fields of AI and echocardiography was conducted on November 10, 2025, retrieving data from inception until the search date using the Scopus (Elsevier) and WoSCC (Clarivate Analytics) databases. The Clarivate WoSCC is a leading database known for rigorous indexing standards and extensive coverage of high-impact scholarly journals [11], whereas the Scopus database offers broader coverage of scholarly journal articles, conference proceedings, books, and preprints [12]. Scopus and WoSCC were combined to improve coverage and reduce database-specific retrieval bias, which is particularly relevant in AI research given the prominence of conference papers and technical publications.

**Fig. 1:**
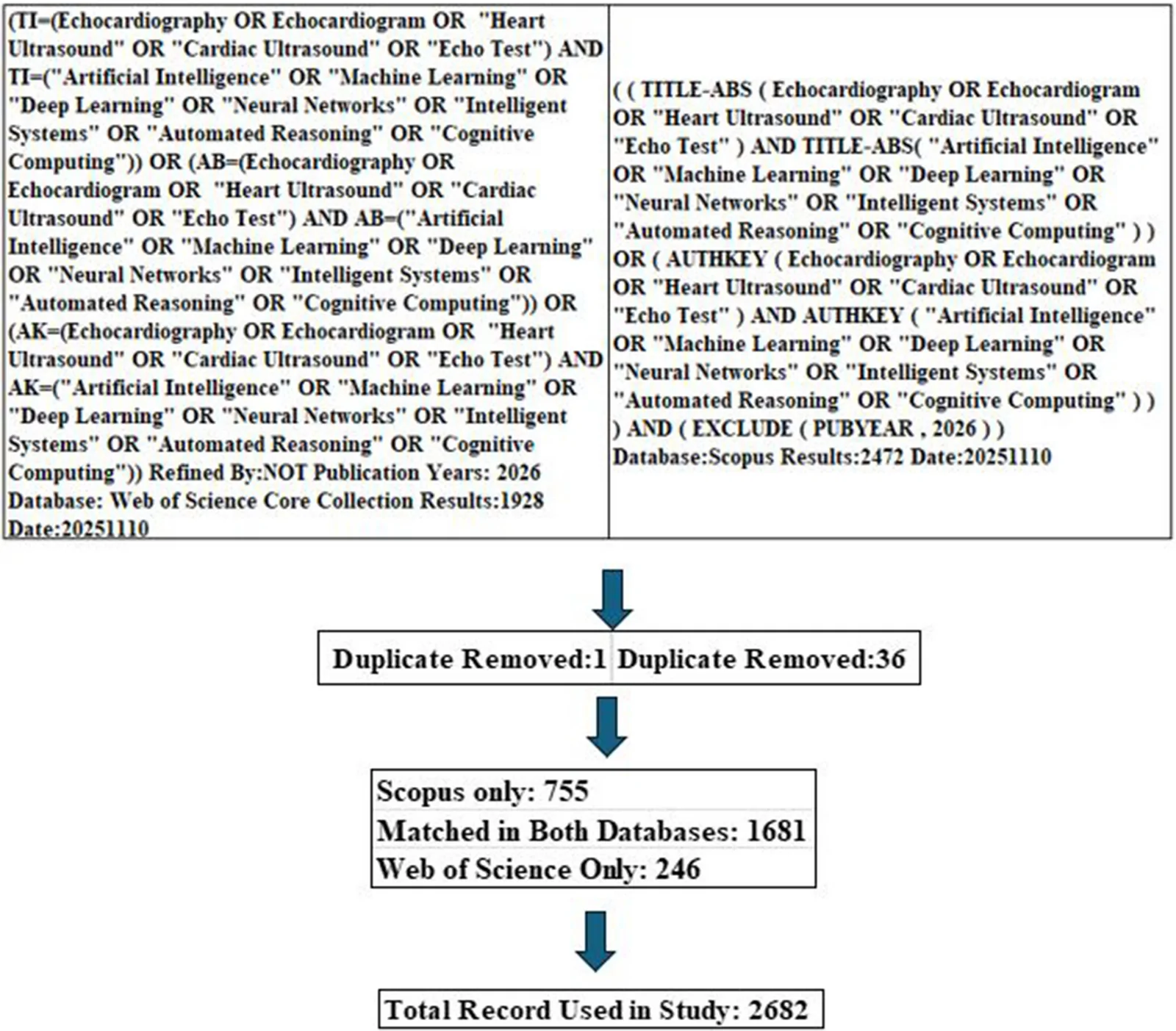
Search strategy flow diagram showing databases queried, keywords used, and records included in this study. The flow diagram shows database searches in Scopus and the Web of Science, duplicate removal, and final study selection. A total of 2,682 publications were retained for final analysis

A comprehensive list of topic-related keywords was combined with Boolean operators to develop the search strategy and formulate the query (Fig. 1). To ensure comprehensive coverage of research contributing to the field, all document types were considered, including original articles, conference papers, review articles, meeting abstracts, books, book chapters, notes, letters, proceedings, editorials, corrections, and retractions. No language filters were applied at the search stage, as complete bibliometric metadata, including titles and abstracts, was available in English for all records regardless of original publication language.

The search returned 1,928 documents from WoSCC and 2,472 from Scopus. A duplicate check was performed using Microsoft Excel (Microsoft Corp) for document titles, authors, and years, which identified 1 duplicate record in the WoSCC results and 36 in the Scopus results; 1,927 and 2,436 records were therefore retained from WoSCC and Scopus, respectively. An analysis of both databases revealed 1,681 records in common; for these overlapping records, Scopus citation counts were retained to avoid double-counting. In total, 755 and 246 unique records were found in Scopus and WoSCC, respectively, and 2,682 records were retained for the final analysis (Fig. 1). The merged dataset therefore combines citation counts from two sources (Scopus for 2,436 records, 90.8%; WoSCC for 246 records, 9.2%). Of the retained records, 96.9% were in English, with the remainder distributed across seven other languages (Table S1).

### Data analysis

Data analysis was performed using Microsoft Excel and Microsoft Access (Microsoft Corp) and IBM SPSS ver. 21 (IBM Corp). Descriptive statistics were used to summarize annual publication output, total citations, citations per publication (C/P), document types, countries, institutions, journals, and keywords; C/P was calculated by dividing the total number of citations by the number of publications in each category. Correlations were assessed using Spearman rank correlation. Citation counts were compared between two groups (open access, funding, and authorship) using the Mann–Whitney U-test, and among three groups (database indexing) using the Kruskal–Wallis test. A sensitivity analysis was performed after excluding the 246 WoSCC-only records, restricting the analysis to records with Scopus citation counts (*n* = 2,436). These comparisons were exploratory and unadjusted for potential confounders. A *P*-value of < 0.05 was considered statistically significant.

Bibliometric mapping and data visualization were performed using Biblioshiny ver. 5.2.0 (K-Synth), Microsoft Power BI (Microsoft Corp), RawGraphs.io (DensityDesign Research Lab, Politecnico di Milano), Visual Basic for Applications (Microsoft Corp), and Word Art (WordArt.com). The country collaboration network was visualized as a chord diagram generated from co-authorship metadata. Arc length reflected each country’s total collaboration link strength (the sum of its co-authored publications with other countries), and ribbon width reflected the number of co-authored publications between country pairs.

The 50 most frequent author keywords were manually assigned to 10 thematic clusters based on conceptual similarity. Keywords were analyzed as recorded without synonym normalization. Thematic evolution was analyzed in Biblioshiny using author keywords, comparing two periods: 1984–2019 and 2020–2025. Themes were linked between periods by shared keywords, and band thickness shows how often these keywords occurred.

Together, these tools enabled systematic data processing and visualization of publication trends, authorship patterns, collaboration networks, research hotspots, and emerging directions in the field.

## Results

### Publication trends

Table 1 summarizes the final records included in the analysis. A total of 2,682 publications related to AI and echocardiography and published between 1984 and 2025 were identified. These publications had 35,083 citations with an overall average of 13.08 C/P. Original articles ranked first in both volume (*n* = 1,509, 56.3%) and citations (27,019 citations, 17.91 C/P), followed by conference papers (*n* = 534, 19.9%; 5.48 C/P), and review articles (*n* = 300, 11.2%; 15.34 C/P) (Table 1). Review articles accounted for 13.1% of total citations versus only 11.2% of publications, a higher share of citations relative to their publication output. Collectively, original articles, conference papers, and review articles accounted for 87.4% and 98.5% of total publications and citations, respectively. The remaining gray literature, including meeting abstracts, editorials, and letters, contributed minimally to the overall citation metrics (Table 1).

**Table 1:** Final records included in this study

**Document type**	No. of total **publications**	No. of total **citations**	No. of citations per **publication**
Original article	1,509	27,019	17.91
Conference paper	534	2,924	5.48
Review	300	4,601	15.34
Meeting abstract	126	103	0.82
Book chapter	58	57	0.98
Editorial	58	193	3.33
Conference review	55	0	0
Letter	17	89	5.24
Note	9	36	4
Erratum	7	2	0.29
Retracted	6	34	5.67
Short survey	1	4	4
Correction	1	0	0
Book	1	21	21
Grand total	2,682	35,083	13.08

**Fig. 2:**
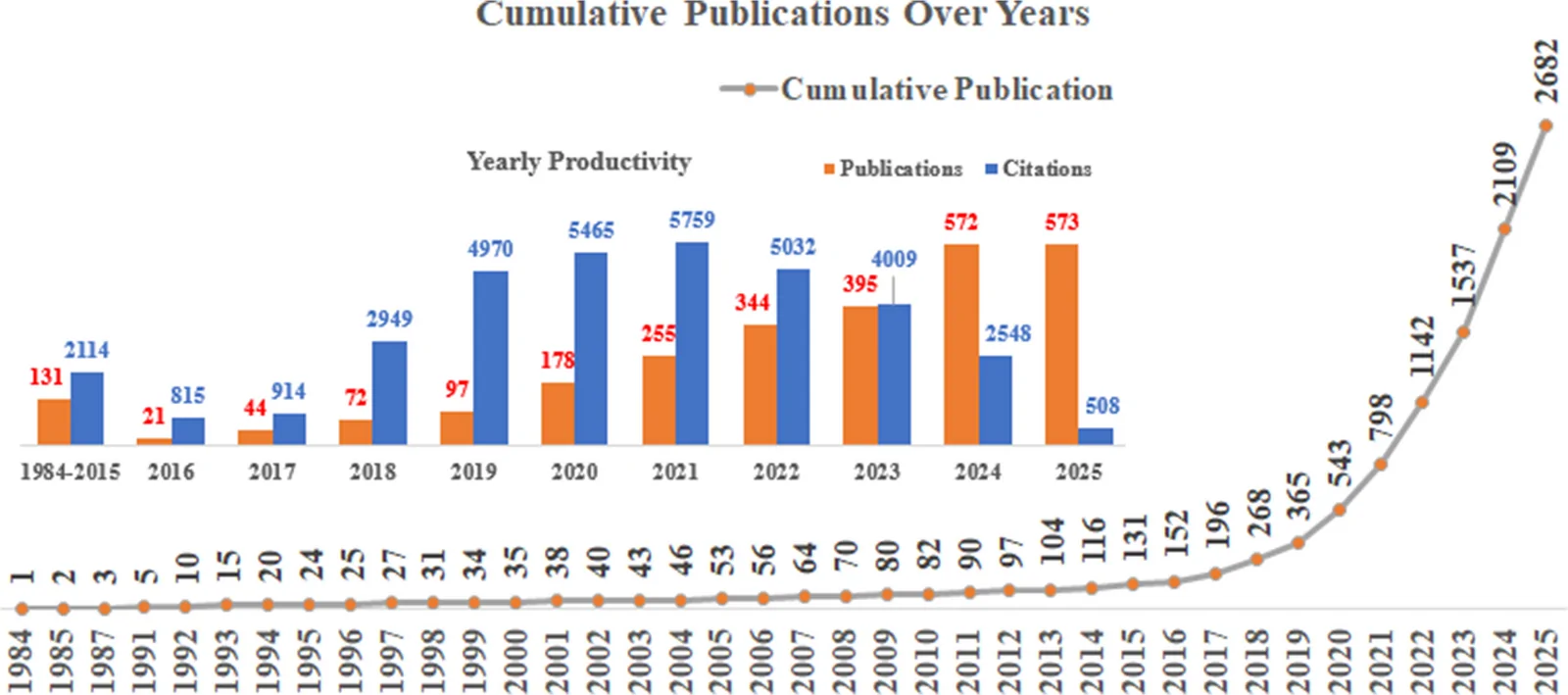
Annual (bar graph) and cumulative (line graph) publication output in artificial intelligence (AI)-echocardiography research. The line graph shows cumulative publications. The bar graph shows annual publications and the citations accrued by each publication-year cohort in AI-echocardiography research from 1984 to 2025. The 2025 data were incomplete at the time of retrieval (November 10, 2025)

Figure 2 shows the annual and cumulative publication output, with the citations received by each publication-year cohort. The research output remained limited until approximately 2015, after which the trend gradually increased before accelerating markedly after 2018. The most notable expansion occurred between 2020 and 2025; annual publications were nearly identical in 2024 (*n* = 572) and 2025 (*n* = 573), although 2025 was observed only until November 10, so the full-year total is likely to be higher. Collectively, these patterns indicate a transition from low initial output to sustained and high research productivity.

Citation trends showed a similar pattern to publication trends, with minimal citations in earlier cohorts followed by a sharp increase after 2018. Citations peaked for the 2021 publication cohort, reflecting an increase in the visibility of research output. The lower citation counts for more recent cohorts reflect their shorter citation window rather than declining citation activity.

### Country and region contributions

**Fig. 3:**
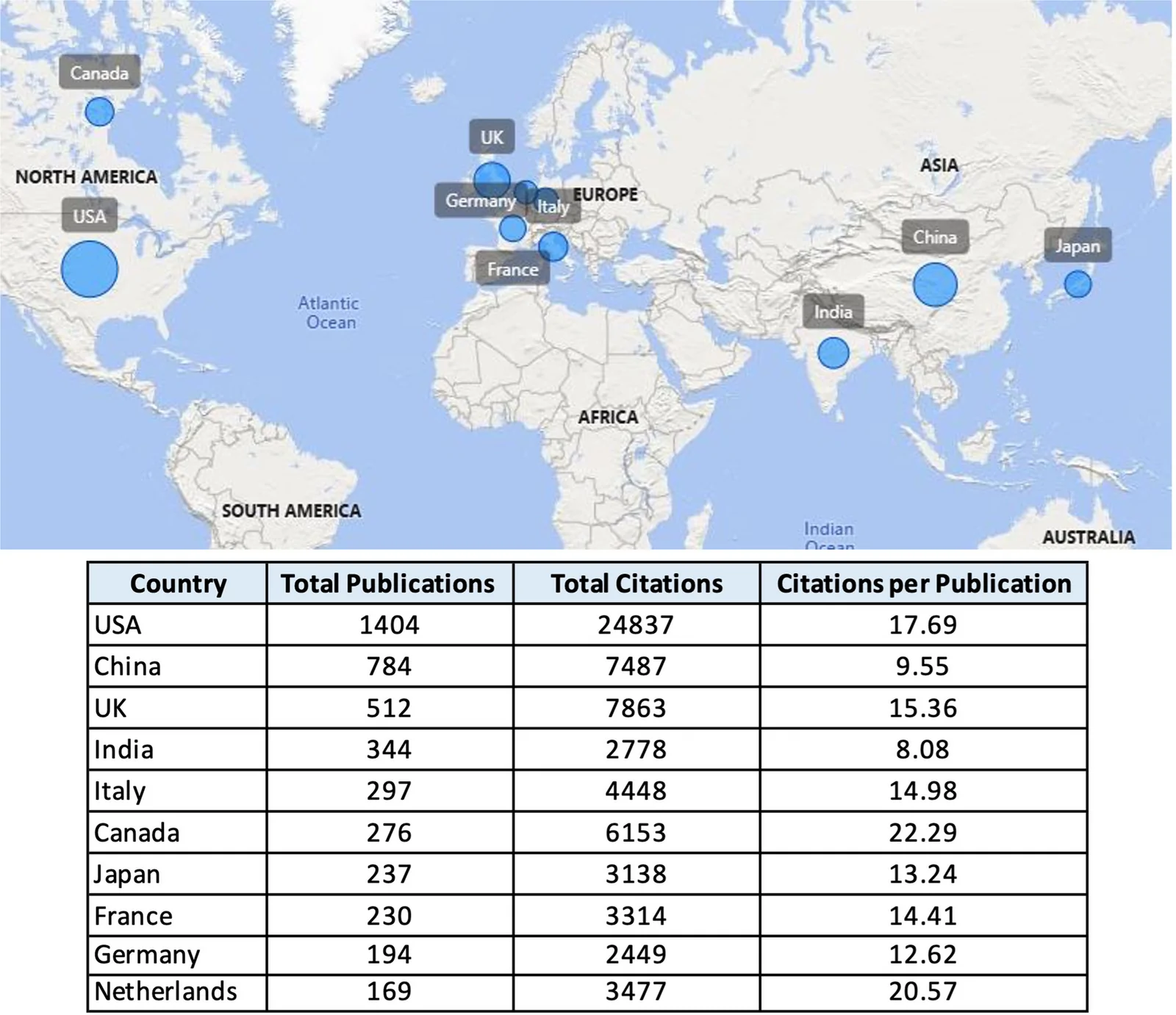
Top contributing countries to artificial intelligence (AI)-echocardiography research and citations. World map shows the ten most productive countries. Bubble size indicates publication output; the table summarizes publications, citations, and citations per publication (generated using Microsoft Power BI, Microsoft Corp)

The top 10 countries/regions with the greatest contribution to literature on AI and echocardiography indexed in Scopus and WoSCC during the study period are shown in Fig. 3, where the publication output of each country is shown as bubbles on a world map, with larger bubbles indicating higher productivity. The United States led in both publication volume and total citations (1,404 publications and 24,837 citations), followed by China (784 publications and 7,487 citations) and the United Kingdom (512 publications and 7,863 citations). Although the United Kingdom produced 35% fewer publications than China, its C/P was considerably higher (15.36 vs. 9.55). Two countries had higher C/P than the United States, despite producing fewer publications: Canada ranked sixth in publication output but first in C/P, and the Netherlands ranked tenth in publication output but second in C/P. This disparity between publication volume and citation count highlights differences between research productivity and citation attention. Variations in journal impact factors, international co-authorship patterns, thematic research priorities, and institutional research maturity may account for these results.

**Fig. 4:**
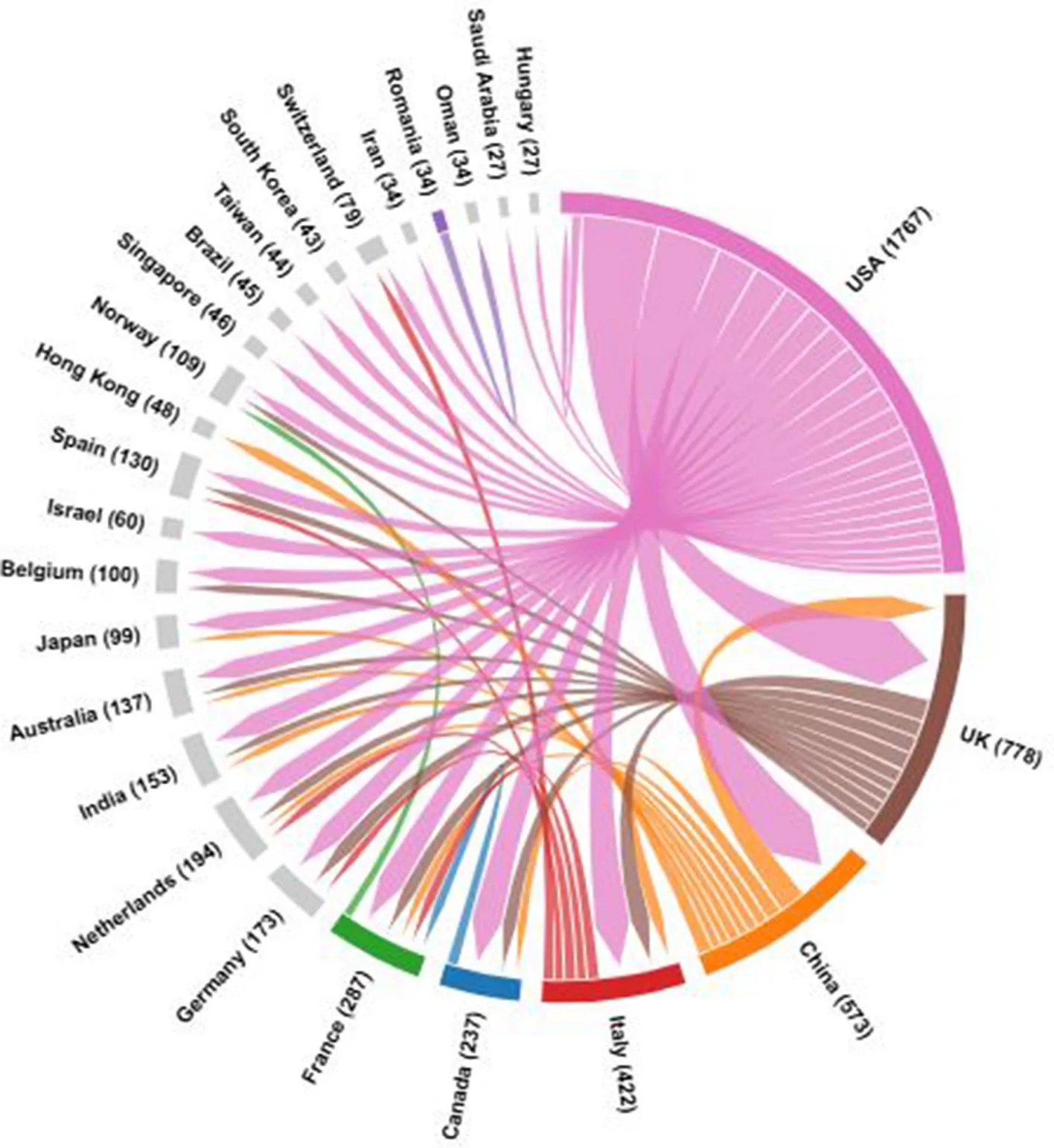
Global collaboration among countries/regions in artificial intelligence (AI)-echocardiography research. Chord diagram illustrates international research collaborations among countries publishing AI-echocardiography studies between 1984 and 2025 (generated using RawGraphs.io, DensityDesign Research Lab, Politecnico di Milano)

Figure 4 shows international collaboration among countries/regions in the field of AI-echocardiography during the study period. The United States had the highest total collaboration link strength (*n* = 1,767), followed by the United Kingdom (*n* = 778) and China (*n* = 573). The United States–United Kingdom collaboration produced the greatest number of co-authored publications (*n* = 237), followed by United States–China (*n* = 199), United States–Italy (*n* = 128), United States–Canada (*n* = 111), United States–France (*n* = 92), and China–United Kingdom (*n* = 87) collaborations. These results indicate that the United States is a major contributor to global research output and collaboration.

### Institutional analysis

The top 10 institutions contributing to the literature on AI and echocardiography indexed in WoSCC and Scopus during the study period are listed in Table 2. Seven were in North America (six in the United States and one in Canada) and three in Europe (two in the United Kingdom and one in Norway). The Mayo Clinic ranked first in both publications (*n* = 142) and citations (*n* = 3,604), followed by Harvard University in publications (*n* = 57). The University of California, Stanford University and the University of Oxford each produced 52 publications, but their citations differed considerably (3,087, 2,458 and 971 citations, respectively). The remaining institutions were the University of British Columbia (45 publications), Yale University (44 publications), the Norwegian University of Science and Technology (44 publications), Cedars-Sinai Medical Center (43 publications), and King’s College London (40 publications), with varying citation counts.

**Table 2:** Top 10 contributing/publishing institutions in literature on AI and echocardiography

Rank	Institution	Country	No. of total publications	No. of total citations	No. of citations per publication	Start year
1	Mayo Clinic	USA	142	3,604	25.38	1992
2	Harvard University	USA	57	1,838	32.25	2011
3	University of California	USA	52	3,087	59.37	2017
4	Stanford University	USA	52	2,458	47.27	2017
5	University of Oxford	UK	52	971	18.67	2009
6	University of British Columbia	Canada	45	1,226	27.24	2017
7	Yale University	USA	44	1,058	24.05	2009
8	Norwegian University of Science and Technology	Norway	44	698	15.86	2017
9	Cedars-Sinai Medical Center	USA	43	2,014	46.84	2019
10	King's College London	UK	40	538	13.45	2017

*AI* artificial intelligence

C/P varied considerably among institutions. The University of California had the highest C/P (59.37), followed by Stanford University (47.27) and Cedars-Sinai Medical Center (46.84). Harvard University had a C/P of 32.25, and the University of British Columbia 27.24. Mayo Clinic, despite the highest publication output, had a moderate C/P (25.38). The University of Oxford (18.67), the Norwegian University of Science and Technology (15.86) and King’s College London (13.45) had lower C/P than the North American institutions. This variability may reflect institutional differences in research focus, publication venue selection and international collaboration intensity.

The first indexed publication year varied among institutions. Among the top 10 institutions, Mayo Clinic had the earliest indexed publication (1992), followed by Yale University and the University of Oxford (both 2009) and Harvard University (2011). Five institutions had their earliest indexed publication in 2017: the University of California, Stanford University, the University of British Columbia, the Norwegian University of Science and Technology, and King’s College London. Cedars-Sinai Medical Center followed in 2019. This clustering coincided with advances in deep learning algorithms and computing infrastructure. However, first indexed publication may not reflect the actual onset of research activity.

### Journal analysis

The top 10 journals publishing literature on AI and echocardiography indexed in Scopus and WoSCC during the study period are summarized in Table 3. The journal scope was diverse, including computer science, cardiology, and multidisciplinary research. *Lecture Notes in Computer Science* led in publication count (*n* = 130), followed by *Circulation* (*n* = 66), *Frontiers in Cardiovascular Medicine* (*n* = 59), *JACC: Cardiovascular Imaging* (*n* = 52), and the *Journal of the American College of Cardiology* (*n* = 49).

**Table 3:** Top 10 sources/journals publishing literature on AI and echocardiography

**Rank**	**Source/Journal**	No. of total publications	No. of total citations	No. of citations per publication	**Start year**
1	*Lecture Notes in Computer Science*^a^	130	821	6.32	1996
2	*Circulation*	66	1,266	19.18	1995
3	*Frontiers in Cardiovascular Medicine*	59	597	10.12	2020
4	*JACC: Cardiovascular Imaging*	52	1,911	36.75	2013
5	*Journal of The American College of Cardiology*	49	1,003	20.47	2015
6	*Journal of The American Society of Echocardiography*	46	778	16.91	2019
7	*European Heart Journal—Digital Health*	41	376	9.17	2021
8	*Journal of Clinical Medicine*	39	363	9.31	2020
9	*European Heart Journal*	39	766	19.64	2017
10	*Scientific Reports*	36	337	9.36	2022

*AI* artificial intelligence

^a^Book series

The citation metrics of the journals were also heterogeneous. Among the top 10 journals, *JACC: Cardiovascular Imaging* ranked first in both total citations (*n* = 1,911) and C/P (36.75). *Circulation* (1,266 citations, 19.18 C/P), the *Journal of the American College of Cardiology* (1,003 citations, 20.47 C/P), and the *European Heart Journal* (766 citations, 19.64 C/P) followed. The *Journal of the American Society of Echocardiography* had 778 citations (16.91 C/P). Recent open access journals, including *Frontiers in Cardiovascular Medicine* (597 citations, 10.12 C/P), *European Heart Journal–Digital Health* (376 citations, 9.17 C/P), the *Journal of Clinical Medicine* (363 citations, 9.31 C/P), and *Scientific Reports* (337 citations, 9.36 C/P), had lower C/P, possibly reflecting their shorter citation accrual time.

### Authorship patterns and author productivity

The top 10 authors (with affiliations) contributing to the literature on AI and echocardiography indexed in Scopus and WoSCC during the study period are summarized in Table 4. Author-level productivity analysis showed that most were based in the United States (8 out of 10), with Mayo Clinic-affiliated authors constituting the majority of the leading contributors (6 out of 10). Francisco Lopez-Jimenez led in publication volume (*n* = 55), followed closely by Zachi I Attia (*n* = 54) and Paul Andrew Friedman (*n* = 52). David Ouyang from the Cedars-Sinai Medical Center ranked fourth (*n* = 47) and Partho P Sengupta from West Virginia University ranked fifth (*n* = 43).

**Table 4:** Top 10 authors publishing literature on AI and echocardiography

**Author**	**Affiliation**	No. of total publications	No. of total citations	No. of citations per publication	**Start year**
Lopez-Jimenez, Francisco	Mayo Clinic, USA	55	2,673	48.6	2019
Attia, Zachi I	Mayo Clinic, USA	54	2,681	49.65	2019
Friedman, Paul Andrew	Mayo Clinic, USA	52	2,662	51.19	2019
Ouyang, David	Cedars-Sinai Medical Center, USA	47	1,976	42.04	2019
Sengupta, Partho P	West Virginia University, USA	43	1,655	38.49	2008
Lovstakken, Lasse	NTNU, Norway	41	1,344	32.78	2017
Tsang, Teresa S M	Mayo Clinic, USA	40	695	17.38	2017
Abolmaesumi, Purang	University of British Columbia, Canada	39	676	17.33	2017
Noseworthy, Peter A	Mayo Clinic, USA	36	2,564	71.22	2019
Pellikka, Patricia A	Mayo Clinic, USA	34	1,769	52.03	2019

*AI* artificial intelligence

Authors with high total citation counts and C/P included Zachi I Attia (2,681 citations, 49.65 C/P), Francisco Lopez-Jimenez (2,673 citations, 48.6 C/P), and Paul Andrew Friedman (2,662 citations, 51.19 C/P). Peter A Noseworthy had the highest C/P (71.22) despite a lower publication output (36 publications, 2,564 citations), followed by Patricia A Pellikka (34 publications, 1,769 citations, 52.03 C/P). Other authors included David Ouyang (1,976 citations, 42.04 C/P) and Partho P Sengupta (1,655 citations, 38.49 C/P).

The first indexed publication year varied among authors. Among the top 10 authors, Partho P Sengupta had the earliest indexed publication (2008), followed by three authors in 2017 (Lasse Lovstakken, Teresa S M Tsang, and Purang Abolmaesumi). The remaining six authors, including five Mayo Clinic investigators, had their first indexed publication in 2019. This clustering coincided with advances in deep learning technology and cardiovascular imaging, although first indexed publication may not reflect the actual onset of research activity.

The authorship patterns of publications on AI and echocardiography identified in this study are shown in Fig. S1. Among the 2,682 total publications, most were products of small collaborative groups of authors, with a peak in output observed among publications by three to six authors (*n* = 1,060, 39.5%). The number of publications gradually decreased with increasing group size, with only a few involving large author groups. Furthermore, only 124 (4.6%) of all publications involved a single author.

### Top-cited publications

The top 10 most cited publications in the field of AI-echocardiography indexed in Scopus and WoSCC during the study period are listed in Table 5 [5, 13–21]. The publication “Fully automated echocardiogram interpretation in clinical practice: feasibility and diagnostic accuracy” by Zhang et al. [5] had the most citations (*n* = 737), followed by “Video-based AI for beat-to-beat assessment of cardiac function” by Ouyang et al. [13] and “Deep learning for segmentation using an open large-scale dataset in 2D echocardiography” by Leclerc et al. [14]. Ouyang et al. had the highest citation frequency (121.5 citations per year), compared with Zhang et al. (92.13 citations per year) and Leclerc et al. (87.29 citations per year), despite comparable total citation counts across these publications. Publications titled “Fast and accurate view classification of echocardiograms using deep learning” by Madani et al. [15] and “Clinical applications of machine learning in cardiovascular disease and its relevance to cardiac imaging” by Al’Aref et al. [16] ranked as the fourth and fifth most highly cited publications, respectively. Other highly cited publications included those by Ghorbani et al. [17], Narula et al. [18], Litjens et al. [19], Narang et al. [20], and Knackstedt et al. [21].

**Table 5:** Top 10 most cited publications in the field of AI–echocardiography

Rank	Bibliographic information	No. of total citations	No. of citations per year	Reference
1	Zhang J, Gajjala S, Agrawal P, Tison GH, Hallock LA, Beussink-Nelson L, et al. Fully automated echocardiogram interpretation in clinical practice. Circulation. 2018;138:1623–35	737	92.13	[5]
2	Ouyang D, He B, Ghorbani A, Yuan N, Ebinger J, Langlotz CP, et al. Video-based AI for beat-to-beat assessment of cardiac function. Nature. 2020; 580:252–6	729	121.5	[13]
3	Leclerc S, Smistad E, Pedrosa J, Ostvik A, Cervenansky F, Espinosa F, et al. Deep learning for segmentation using an open large-scale dataset in 2D echocardiography. IEEE Trans Med Imaging. 2019;38:2198–210	611	87.29	[14]
4	Madani A, Arnaout R, Mofrad M, Arnaout R. Fast and accurate view classification of echocardiograms using deep learning. NPJ Digit Med. 2018;1:6	453	56.63	[15]
5	Al'Aref SJ, Anchouche K, Singh G, Slomka PJ, Kolli KK, Kumar A, et al. Clinical applications of machine learning in cardiovascular disease and its relevance to cardiac imaging. Eur Heart J. 2019;40:1975–86	390	55.71	[16]
6	Ghorbani A, Ouyang D, Abid A, He B, Chen JH, Harrington RA, et al. Deep learning interpretation of echocardiograms. NPJ Digit Med. 2020; 3:10	370	61.67	[17]
7	Narula S, Shameer K, Salem Omar AM, Dudley JT, Sengupta PP. Machine-learning algorithms to automate morphological and functional assessments in 2D echocardiography. J Am Coll Cardiol. 2016;68:2287–95	341	34.1	[18]
8	Litjens G, Ciompi F, Wolterink JM, de Vos BD, Leiner T, Teuwen J, et al. State-of-the-art deep learning in cardiovascular image analysis. JACC Cardiovasc Imaging. 2019;12:1549–65	319	45.57	[19]
9	Narang A, Bae R, Hong H, Thomas Y, Surette S, Cadieu C, et al. Utility of a deep-learning algorithm to guide novices to acquire echocardiograms for limited diagnostic use. JAMA Cardiol. 2021;6:624–32	288	57.6	[20]
10	Knackstedt C, Bekkers SC, Schummers G, Schreckenberg M, Muraru D, Badano LP, et al. Fully automated versus standard tracking of left ventricular ejection fraction and longitudinal strain: the FAST-EFs multicenter study. J Am Coll Cardiol. 2015;66:1456–66	216	19.64	[21]

*AI* artificial intelligence

Many of the top 10 most cited publications were published between 2018 and 2020 (n = 7), which coincided with the exponential growth phase shown in Fig. 2. Notably, pre − 2015 works were absent from the top 10 highly cited publications, consistent with the increase in deep learning-based research in recent years.

### Keyword occurrence analysis

The top 50 author keywords used in the literature on AI and echocardiography indexed in Scopus and WoSCC during the study period are summarized in Table 6. The most frequently used keywords were “echocardiography” (*n* = 872), “deep learning” (*n* = 615), “artificial intelligence” (*n* = 605), and “machine learning” (*n* = 489).

**Table 6:** Top 50 author keywords used in the literature on AI and echocardiography

Keyword	No. of occurrences	Thematic cluster
**Echocardiography**	872	Core imaging modality
**Deep** learning	615	AI/ML methodology
**Artificial** intelligence	605	AI/ML methodology
**Machine** learning	489	AI/ML methodology
**Heart** failure	128	Cardiac function & anatomy
**Echocardiogram**	82	Core imaging modality
**Segmentation**	82	Computational tasks
**Electrocardiogram**	71	Multimodal integration
**Convolutional** neural network	68	AI/ML methodology
**Ultrasound**	67	Core imaging modality
**Ejection** fraction	60	Cardiac function & anatomy
**Aortic** stenosis	59	Valvular pathology
**Electrocardiography**	57	Multimodal integration
Congenital heart disease	53	Structural & congenital
Classification	50	Computational tasks
**Left** ventricle	45	Cardiac function & anatomy
**Atrial** fibrillation	44	Arrhythmia
**Fetal** echocardiography	42	Core imaging modality
**Image** segmentation	42	Computational tasks
**Neural** networks	37	AI/ML methodology
**Left** ventricular ejection fraction	36	Cardiac function & anatomy
**Hypertrophic** cardiomyopathy	34	Structural & congenital
**Neural** network	34	AI/ML methodology
**Cardiovascular** imaging	33	General cardiovascular
**Coronary** artery disease	32	Ischemic & vascular
**Cardiovascular** disease	31	General cardiovascular
**Convolutional** neural networks	31	AI/ML methodology
**Mitral** regurgitation	29	Valvular pathology
**Myocardial** infarction	28	Ischemic & vascular
**Valvular** heart disease	28	Valvular pathology
**Diagnosis**	27	Computational tasks
**Cardiac** imaging	26	General cardiovascular
**Cardiology**	26	General cardiovascular
**Transesophageal** echocardiography	26	Core imaging modality
**Cardiomyopathy**	25	Structural & congenital
**Cardiovascular** diseases	25	General cardiovascular
**Computed** tomography	25	Multimodal integration
**Prognosis**	25	Computational tasks
**Transthoracic** echocardiography	25	Core imaging modality
**U-Net**	25	AI/ML methodology
**ECG**	24	Multimodal integration
**Radiomics**	24	AI/ML methodology
**Cardiac** magnetic resonance	23	Multimodal integration
**Medical** imaging	23	General cardiovascular
**Random** forest	23	AI/ML methodology
**Heart** disease	22	General cardiovascular
**Pulmonary** hypertension	22	Ischemic & vascular
**Strain**	22	Cardiac function & anatomy
**Stress** echocardiography	22	Core imaging modality
**Artificial** intelligence **(AI)**	21	AI/ML methodology

*AI* artificial intelligence, *ECG* electrocardiogram, *ML* machine learning

Keyword occurrence analysis, grouping 50 keywords with 4,365 total occurrences, yielded 10 thematic clusters (Table 6). The two largest clusters, AI/machine learning (ML) methodology (1,972 occurrences, 45.2%) and echocardiography (core imaging modality) (1,136 occurrences, 26.0%), together represented 71.2% of the keyword frequency. The clinically oriented clusters were diverse, including cardiac function and anatomy; valvular pathology; structural and congenital disease; ischemic and vascular disease; and arrhythmia, in addition to a separate core computational tasks cluster, comprising segmentation, classification, diagnosis, and prognosis.

The most represented disease domains by keyword occurrence were heart failure (*n* = 128), valvular heart disease (*n* = 116), coronary artery disease/ischemic heart disease (*n* = 60), cardiomyopathy (*n* = 59), congenital heart disease (*n* = 53), and arrhythmia (*n* = 44) (Table S2). Similarly, the most represented investigative-purpose keywords, drawn from the computational tasks cluster, were segmentation (*n* = 124), classification (*n* = 50), diagnosis (*n* = 27), and prognosis (*n* = 25) (Table S3).

**Fig. 5:**
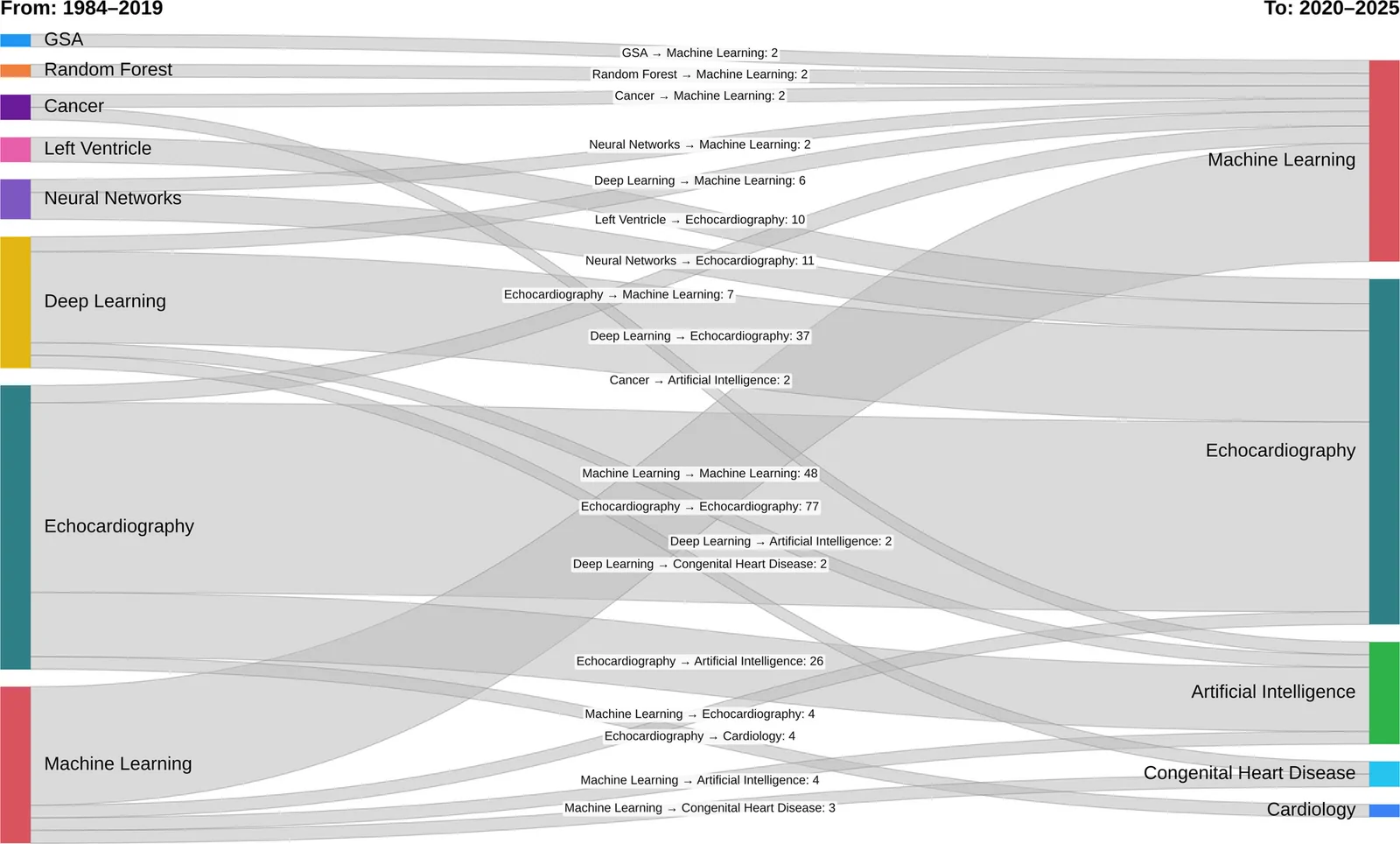
Thematic evolution of artificial intelligence (AI)-echocardiography research topics from 1984–2019 to 2020–2025. Sankey diagram shows the links between major research themes in the two periods. Band thickness represents the occurrences of shared keywords (generated using Python)

Figure 5 shows how the research topics from 1984–2019 evolved into the main themes from 2020–2025, with the band thickness indicating the degree of thematic continuity. On the left, early keywords such as echocardiography, ML, deep learning, neural networks, and left ventricle formed the beginning nodes. Over time, these terms expanded, merged, or redirected. During 2020–2025, research topics consolidated around ML, echocardiography, AI, congenital heart disease, and cardiology. Most flows reflected thematic continuity, with emerging clinically oriented topics.

### Factors associated with citation counts

Publication age was moderately correlated with citation count (Spearman coefficient, 0.50; *P* < 0.001). Open access publications (*n* = 1,283) had higher citation counts than non–open access publications (*n* = 1,399; median [interquartile range (IQR)], 5 [1–16] vs. 1 [0–7]; *P* < 0.001). Funded publications (*n* = 1,240) had higher citation counts than unfunded publications (*n* = 1,442; median [IQR], 4 [1–15] vs. 2 [0–9]; *P* < 0.001). Compared to single-author publications (*n* = 124), multiple-author publications (*n* = 2,558) had higher citation counts (median [IQR], 3 [0–12] vs. 0 [0–2]; *P* < 0.001). Citation counts differed by indexing coverage (*P* < 0.001), being highest for publications indexed in both Scopus and WoSCC (*n* = 1,681; median [IQR], 5 [1–16.5]), followed by those indexed only in Scopus (*n* = 755; median [IQR], 1 [0–5]), and only in WoSCC (*n* = 246; median [IQR], 0 [0–2]). In a sensitivity analysis restricted to records with Scopus citation counts (n = 2,436), all associations remained in the same direction and statistically significant (all *P* < 0.001) (Table S4). As these are unadjusted comparisons, they should not be interpreted as independent associations.

## Discussion

This comprehensive and up-to-date bibliometric analysis of 2,682 publications covering the period 1984–2025 shows that AI-echocardiography research represents a rapidly expanding field, with emerging clinically oriented topics. Since 2018, research productivity has grown rapidly, with similar output in 2024 and 2025 despite incomplete 2025 data. This growth likely reflects advances in deep learning techniques, increased access to large datasets, and improved computational capabilities [22], coinciding with landmark publications in the field of AI-echocardiography [5, 15].

Two recent bibliometric studies have examined AI-echocardiography research: Liu et al. [9] mapped research hotspots using WoSCC records from 2009–2024, and Chen et al. [10] characterized the research landscape and key contributors using WoSCC records from 1997–2024. Both offer valuable WoSCC-based snapshots of the field through 2024. Differences in record counts mainly reflect search strategies and eligibility criteria rather than database number alone (Table 7) [9, 10]. Both studies were limited to English-language articles and reviews; Liu et al. [9] used a broad topic search without relevance screening (3,411 records), whereas Chen et al. [10] applied manual screening (605 records). The present study searched titles, abstracts and author keywords, without language or document-type restrictions (2,682 records). These differences yielded meaningfully different findings. China ranked first in publications in Liu et al. [9], whereas the United States ranked first in Chen et al. [10] and in the present study, and the leading institution was Shanghai Jiao Tong University in Liu et al. [9] (with 6 of their top 10 institutions in China) and Stanford University in Chen et al. [10], compared with Mayo Clinic in the present study. Including conference papers (19.9% of records) identified *Lecture Notes in Computer Science* as the most productive source, a computer science publication stream not captured in article-and-review-only analyses. The longer historical coverage added 70 pre − 2009 records, including Mayo Clinic’s first indexed publication in 1992, and the 2025 records showed output similar to 2024 despite incomplete data, which was not observed in studies ending in 2024.

**Table 7:** Comparison with recent AI-echocardiography bibliometric studies

Feature	Liu et al. [9] (2025)	Chen et al. [10] (2025)	Present Study
Database	WoSCC (SCIE)	WoSCC (SCIE and SSCI)	Scopus and WoSCC
Period	2009–2024	1997–2024	1984–2025
Search fields and terms	Topic field (including Keywords Plus); broad AI and echocardiographic terms (e.g., SVM, random forest, speckle tracking, LVEF)	Topic field (including Keywords Plus); broad AI (e.g., big data, data mining, robotics) and echocardiography terms	Title, abstract and author keywords; AI and echocardiography terms
Language/screening	English only; no relevance screening (3,784 → 3,411 after document-type exclusion)	English only; manual screening (1,612 → 605)	No language limit; deduplication only
Document types	Articles and reviews	Articles and reviews	Broad document set (articles, conference papers, reviews, abstracts, book chapters, editorials, letters, etc.)
No. of records analyzed	3,411	605	2,682
Main emphasis	Research hotspots and visualization	Research landscape and key contributors	Historical trajectory, database overlap, document-type contribution, collaboration structure, exploratory citation-associated factors,

*AI* artificial intelligence, *LVEF* left ventricular ejection fraction, *WoSCC* Web of Science Core Collection, *SCIE* Science Citation Index Expanded, *SSCI* Social Sciences Citation Index, *SVM* support vector machine

The United States has historically led the research field, with the highest research productivity and total citations. Seven of the top 10 contributing institutions are in North America (six in the United States and one in Canada), with Mayo Clinic exemplifying sustained institutional leadership. The availability of large institutional repositories, such as Stanford’s EchoNet-Dynamic (10,030 echocardiographic videos), has enabled the development of generalizable algorithms [13]. Despite having the second-highest publication volume, China’s C/P is considerably lower than that of the United Kingdom, which is ranked third (9.55 vs. 15.36). This disparity may be associated with several nonmutually exclusive factors, including differences in publication age, journal visibility, database coverage, international co-authorship patterns, research focus, and citation practices [16, 23]. However, these explanations remain inferential and should not be interpreted as causal. In international collaboration, the United States has the highest link strength, followed by the United Kingdom and China, with the United States–United Kingdom collaboration producing the most co-authored publications. These patterns suggest that greater investment in international consortia could help address these disparities by promoting dataset sharing and improving algorithmic generalizability across populations, protocols, and equipment [13, 24, 25].

Five of the top 10 institutions (University of California, USA; Stanford University, USA; University of British Columbia, Canada; Norwegian University of Science and Technology, Norway; and King’s College London, UK) had their first indexed publication in 2017. This temporal clustering coincided with the broader expansion of deep learning algorithms and computational infrastructure during this period, although this association should not be interpreted as evidence of direct causation. Moreover, first appearance in the dataset may not reflect the actual onset of institutional research activity. Mirroring the institutional pattern, 8 of the 10 most productive authors in the field were affiliated with institutions in the United States, with contributors from Mayo Clinic representing a notable proportion of the leading researchers. The highest publication output and total citations were observed among the following authors: Francisco Lopez-Jimenez, Zachi I Attia, and Paul Andrew Friedman. Despite producing fewer publications than the leading contributors, Peter A Noseworthy had the highest C/P, followed by Patricia A Pellikka.

AI-echocardiography research from 1984 to 2025 has been historically characterized by peer-reviewed scientific outputs. Original articles, conference papers, and reviews accounted for 87.4% and 98.5% of all indexed publications and citations, respectively. This concentration indicates that growth and citation activity within this field have relied heavily on peer-reviewed publications, which have introduced and evaluated methodological innovations and clinical applications [16, 23, 25]. Among the top journals, *JACC: Cardiovascular Imaging* had the highest total citations and C/P [19, 26–31]. *Circulation*, the *Journal of the American College of Cardiology*, and the *European Heart Journal* also had high C/P [5, 16, 18, 21]. This prominence may reflect the role of specialized cardiovascular journals in reaching clinical audiences. Feasibility and validation studies, such as those by Ouyang et al. [13] and Zhang et al. [5], were among the most cited publications; however, citation counts alone cannot establish whether clinical validation is valued over technical novelty.

Citation trends in this field mirror publication growth, with minimal citations to early publications and a marked increase from 2018, and publications from 2021 receiving the most citations. The lower citation counts for more recent publications reflect their shorter citation window rather than declining citation attention. Several factors were associated with citation counts: publications with longer citation windows tended to accumulate more citations; open access publications and funded research outputs received higher citation counts than non–open access and unfunded research outputs; publications with multiple authors received more citations than single-author publications, which may reflect the broader dissemination associated with collaborative networks; and citation counts varied with indexing coverage, with publications indexed in both Scopus and WoSCC achieving more citations than those indexed in only one database.

Among the 50 most frequently used author keywords characterizing the AI-echocardiography research landscape, the most common were “echocardiography,” “deep learning,” “artificial intelligence,” and “machine learning.” Among the 10 thematic clusters, the two largest clusters were AI/ML methodology and echocardiography as the core imaging modality. The thematic evolution showed continuity of algorithmic-centric themes (1984–2019) with emerging clinically oriented themes (2020–2025). However, these keyword patterns reflect topics rather than clinical implementation.

Future research in the field of AI-echocardiography should move beyond incremental algorithmic improvements and retrospective single-center model development and prioritize robust multicenter validation and prospective clinical implementation studies across diverse populations, disease types, and equipment platforms [32–34]. Key areas may include external validation across scanners and populations, transparent reporting of model performance, and explainable AI methods to improve clinician trust and support clinical adoption. For equitable adoption of this technology, the global disparities in research output identified in this study should be addressed, clinical-computational integration should be enhanced, and algorithmic fairness should be ensured. Given the rapid expansion of the field, the focus should shift toward quality-oriented evaluation of research outputs [35]. Funding bodies, journals, and institutions should prioritize robust clinical validation, reproducibility, dataset transparency, and real-world implementation over novelty claims [35]. Furthermore, future research should also capture real-world impact beyond citations, including but not limited to regulatory approvals, commercial adoption, and clinical guideline integration [36]. Generative and foundation model approaches represent a promising future direction, potentially integrating echocardiographic imaging with clinical and electronic health record data for tasks such as automated structured reporting and decision support. However, challenges in verification, privacy, and bias underscore the need for rigorous validation and human oversight before clinical adoption [37].

This study benefited from including records from two major databases and extending the search period to provide a comprehensive and historical overview of AI applications in echocardiography. However, it also had several limitations. First, relevant publications from other databases, such as PubMed/MEDLINE, or nonindexed journals, may have been excluded, which could limit the comprehensiveness of the analysis. Second, although no language filter was applied during the search, retrieved publications were predominantly in English. This skew likely reflects the language coverage of the databases searched rather than a deliberate exclusion, but it may still mean that relevant non-English studies, particularly from countries with significant AI research activity, were underrepresented in the retrieved records, potentially underestimating their contributions to the field. The observed predominance of the United States may therefore partly reflect database indexing preferences, with non-Western research possibly underrepresented owing to language restrictions or journal selection biases. Third, bibliometric studies are vulnerable to publication bias, as positive and novel AI studies are more likely to be published and cited, whereas negative validation studies, failed implementation studies, and local quality-improvement projects may be underrepresented. Fourth, bibliometric analysis can identify associations, patterns, and trends, but cannot establish causality. Bibliometric metrics, including citation counts, reflect scholarly visibility rather than research quality, methodological rigor, clinical validity, or translational impact, as they are also shaped by publication age, journal visibility, self-citation practices, and database coverage. Retaining Scopus citation counts for overlapping records may introduce additional database-specific bias. Fifth, this study did not perform full co-citation analysis, bibliographic coupling, citation-burst detection, or independent cluster-validation metrics; these methods could offer deeper insight into the intellectual structure and knowledge foundations but require harmonized cited-reference data across databases. Finally, the analysis included literature until November 10, 2025; therefore, 2025 data were incomplete and the most recent research was not captured. Nonetheless, this comprehensive analysis provides valuable insights into the historical evolution, key contributors, and emerging trends of AI-echocardiography research.

## Conclusions

This comprehensive, up-to-date bibliometric analysis of 2,682 publications covering the 1984–2025 period highlights AI-echocardiography research as a rapidly expanding field with emerging clinically oriented topics. The current research landscape is characterized by rapid growth since 2018, institutional predominance of the United States, and sustained leadership by Mayo Clinic, a pattern also reflected at the author level. Future research should prioritize equitable adoption, clinical-computational integration, dataset transparency, reproducibility, robust prospective study designs, and assessment of real-world impact beyond citation metrics, including regulatory approvals and clinical guideline integration. The future success of AI-echocardiography will ultimately depend on rigorous validation, real-world effectiveness, and meaningful improvements in patient outcomes.

## Supplementary Information

Additional file 1: Fig. S1. Authorship patterns of literature on AI and echocardiography identified in this study. Table S1. Language distribution of records retrieved from Scopus and Web of Science Core Collection. Table S2. Disease-domain distribution of author keywords across the included AI-echocardiography publications. Table S3. Investigative-purpose distribution of author keywords across the included AI-echocardiography publications. Table S4. Sensitivity analysis of citation-associated factors restricted to records with Scopus citation counts

## Data Availability

The data that support the findings of this study are included in the article. Further inquiries can be directed to the corresponding author.

## Abbreviations

AI: Artificial intelligence
ML: Machine learning
C/P: Citations per publication
WoSCC: Web of Science Core Collection
