# Supplementary material for: Global research trends of artificial intelligence in echocardiography (1984–2025): a bibliometric analysis
Source: J Cardiovasc Imaging. 2026 Nov 10;34:45. doi: 10.1186/s44348-026-00106-4 (PMC13749735; doi:10.1186/s44348-026-00106-4)
Supplement: Supplementary file 1 [file 44348_2026_106_MOESM1_ESM.docx]

**Fig. S1: Authorship patterns of literature on AI and echocardiography identified in this study.**

Bar chart shows the distribution of publications according to the number of authors per article.

| **Table S1 Language distribution of records retrieved from Scopus and Web of Science Core Collection** | | |
| --- | --- | --- |
| **Languages** | **Publications (n)** | **Percentage (%)** |
| **English** | 2600 | 96.9 |
| **Chinese** | 46 | 1.7 |
| **Russian** | 17 | 0.6 |
| **German** | 7 | 0.3 |
| **Turkish** | 4 | 0.1 |
| **Italian** | 3 | 0.1 |
| **Spanish** | 3 | 0.1 |
| **Ukrainian** | 2 | 0.1 |
| **Total** | 2682 | 100.0 |

*Note: No language filter was applied during the search; this distribution reflects the languages of all records retained for the final analysis.*

| **Table S2** Disease-domain distribution of author keywords across the included AI-echocardiography publications | | |
| --- | --- | --- |
| **Disease Domain** | **Author Keywords Included** | **n** |
| Heart Failure | Heart Failure | 128 |
| Valvular Heart Disease | Aortic Stenosis, Mitral Regurgitation, Valvular Heart Disease | 116 |
| Coronary Artery Disease/Ischemic Heart Disease | Coronary Artery Disease, Myocardial Infarction | 60 |
| Cardiomyopathy | Hypertrophic Cardiomyopathy, Cardiomyopathy | 59 |
| Congenital Heart Disease | Congenital Heart Disease | 53 |
| Arrhythmia | Atrial Fibrillation | 44 |

*Note: Author keywords grouped by disease domain from Table-6; n = combined keyword occurrence, not number of studies.*

| **Table S3** Investigative-purpose distribution of author keywords across the included AI-echocardiography publications | | |
| --- | --- | --- |
| **Investigative Purpose** | **Author Keywords Included** | **n** |
| Segmentation | Image Segmentation, Segmentation | 124 |
| Classification | Classification | 50 |
| Diagnosis | Diagnosis | 27 |
| Prognosis | Prognosis | 25 |

*Note: Author keywords grouped by investigative purpose from Table-6 (“computational tasks” cluster); n = combined keyword occurrence, not number of studies.*

| **Table S4** Sensitivity analysis of citation-associated factors restricted to records with Scopus citation counts | | | | |
| --- | --- | --- | --- | --- |
| **Factor** | **Group** | **Full dataset (n=2682)**  **n; median [IQR]** | **Scopus-only citation source (n=2436)**  **n; median [IQR]** | **p-value (sensitivity analysis)** |
| Publication age |  | Spearman’s coefficient = 0.50 | Spearman’s coefficient = 0.54 | p < 0.001 |
| Open access | Open access | 1283; 5 [1-16] | 1233; 5 [1-16] | p < 0.001 |
|  | Not open access | 1399; 1 [0-7] | 1203; 2 [0-9] |  |
| Funding | Funded | 1240; 4 [1-15] | 1169; 5 [1-16] | p < 0.001 |
|  | Not funded | 1442; 2 [0-9] | 1267; 2 [0-10] |  |
| Authorship | Multiple authors | 2558; 3 [0-12] | 2317; 4 [0-13] | p < 0.001 |
|  | Single author | 124; 0 [0-2] | 119; 0 [0-2] |  |
| Database indexing | Both databases | 1681; 5 [1-16.5] | 1681; 5 [1-16.5] | p < 0.001 |
|  | Scopus only | 755; 1 [0-5] | 755; 1 [0-5] |  |
|  | WoSCC only | 246; 0 [0-2] | Excluded |  |

*Citation counts are presented as median [interquartile range]. The sensitivity analysis excluded the 246 WoSCC-only records, for which WoSCC citation counts were used.*
